# Effect of Bioinductive Cavity Liners on Shear Bond Strength of Dental Composite to Dentin

**DOI:** 10.1155/2022/3283211

**Published:** 2022-03-18

**Authors:** Saba Tohidkhah, Elham Ahmadi, Mahdi Abbasi, Reza Morvaridi Farimani, Ladan Ranjbar Omrani

**Affiliations:** ^1^School of Dentistry, Tehran University of Medical Sciences, Tehran, Iran; ^2^Department of Restorative Dentistry, Tehran University of Medical Science, Tehran, Iran; ^3^Department of Orthodontics, School of Dentistry, Shahid Beheshti University of Medical Sciences, Tehran, Iran; ^4^Dental Research Center, Dentistry Research Institute, Tehran University of Medical Science, Tehran, Iran

## Abstract

**Background:**

The aim of the current study was to evaluate and compare the influence of Dycal, Lime-Lite, TheraCal LC, Biodentine, resin-modified glass ionomer cement (RMGIC), and Activa BioActive as the pulp capping material on the shear bond strength of resin composite to dentin.

**Methods:**

A total of 70 extracted caries-free molars were randomly assigned to seven groups. Six test groups were covered with various protective liners: Dycal, TheraCal LC, Lime-Lite, Activa BioActive, Biodentine, and RMGIC. The control group received no liner pretreatment. Each sample was bonded to resin composite using the total-etch Tetric N bond adhesive. The samples were then tested for shear bond strength using the universal testing machine at a cross-head speed of 1 mm/min until bond failure occurred. The data were analyzed using the one-way ANOVA test followed by the Tamhane post hoc test for pairwise comparisons of the groups.

**Results:**

Independent of the type of the applied liner, all groups exhibited inferior SBS to dentin compared to the control group. TheraCal and RMGIC showed significantly higher shear bond strength than Biodentine and Dycal, which showed the lowest shear bond strength. Lime-Lite and Activa also had significantly lower SBS results than TheraCal. The mode of fracture was predominantly cohesive in Dycal, Biodentine, and TheraCal and adhesive in Activa.

**Conclusion:**

This present study concludes that the bond strength of resin composite to dentin can be affected differently using various types of liners.

## 1. Introduction

Protective dental liners are applied in deep cavities to protect the pulp from different stimuli and facilitate the formation of reparative dentin [1]. These materials can seal dentinal tubules and protect the pulp from microorganisms' attacks and also have therapeutic effects and irritants as well as thermomechanical stimuli [2]. Various materials have been used as cavity liners, including resin-modified glass ionomer cement, calcium silicate-based liners, calcium hydroxide liners, and bioactive glass-based cement [1, 3, 4].

Traditionally, calcium hydroxide has been considered the gold standard of cavity lining materials for several years, in the case of direct and indirect pulp capping treatment procedures due to its excellent antibacterial properties, alkaline pH, and its bioactivity in terms of formation of the hard tissue barrier [5]. However, the unfavorable effects of calcium hydroxide, such as the weak physical properties, tunnel defects, high solubility, and gradual dissolution, led to a decline in its use as a liner with time [6]; to overcome these undesirable drawbacks, several other materials have been introduced; light-activated calcium hydroxide lining materials were one of them which provide improved physical properties and decreased solubility in acids and water, although their bond to the substrate is weak and can shrink during polymerization. [3, 7]. Lime-Lite is a new resin-based light-activated calcium hydroxide-based material that could be applied as a liner and base [8] according to the manufacturer: this material contains hydroxyapatite (HA) and releases hydroxyl, fluoride, and calcium [9].

Tricalcium silica-based cements are other materials launched to the market to compensate for weaknesses of calcium hydroxide-based materials [10]. MTA, Biodentine, and TheraCal LC are among the popular calcium silicate-based liners that can be applied as direct and indirect pulp capping materials [11]. Biodentine is a new liner of this group that can be used as a dentin replacement material under resin composite restorations because it showed comparable mechanical characteristics to dentin [12–14]. When compared with MTA, TheraCal and Biodentine have the better sealing ability, higher compressive strength, lower setting time, and biocompatible and bioactive properties [13, 15, 16].

For deeper restorations that are near to the pulp, without the pulp exposure, an RMGIC is one of the best choices. These materials are dimensionally very stable, release fluoride, and bond to dentin and composite [3, 17]. Furthermore, the adaptation of RMGIC over some liners has been advised; for instance, in order to overcome the drawbacks of calcium hydroxide liners, a protective layer of RMGIC base over this liner is recommended, especially in deeper cavities. To illustrate, RMGIC can act as an insoluble barrier and prevent the microorganism from proceeding toward the pulp at the time when microleakage occurs [4].

Activa BioActive BASE/LINER was introduced by Pulpdent Corporation in 2014. This product is described as an enhanced RMGIC because, besides the properties of an RMGIC, it also has a modified resin matrix with improved physical characteristics [18, 19]. Activa BioActive material reacts to pH changes in the mouth and starts to actively release high amounts of fluoride, calcium, and phosphate to maintain the chemical integrity of the tooth structure; this is how this material plays its bioactive role [20, 21].

Although there are studies asserting that the internal adaptation of resin composite restorations when no liner was applied is more acceptable [22–24], we still need to use them in specific situations; therefore, there should be in vitro experimentations to evaluate their physical properties as a pulp capping material beneath composite restorations because the bond strength of liner and composite as a complex to dentin is of critical importance to the long-term success of a restoration.

For these reasons and also the limited information in the literature about lining material behavior beneath composite restoration, this study was designed to evaluate the effect of these liners on the shear bond strength of the liner-composite complex to dentin.

## 2. Materials and Methods

This in vitro experimental study assessed caries-free human molars (maxillary and mandibular) that had been extracted for purposes other than this study, such as periodontal disease or orthodontic purposes. It is part of the Dental Faculty of the Tehran University of Medical Sciences (TUMS) principles that all patients or their parents for the patients below 18 years old have to be informed and consented to use their extracted teeth for research purposes before extraction at the oral and maxillofacial surgery department and signed an informed consent form for this purpose. The ethics committee of the Dental Faculty of the Tehran University of Medical Sciences approved the study protocols with the code number “IR.TUMS.DENTISTRY.REC.1398.173.”

According to a previous study, with the same methodology [7], the minimum sample size for shear bond strength (SBS) testing was calculated to be 10 in each study group, using one-way ANOVA Power Analysis (SPSS 26), assuming alpha = 0.05, beta = 0.2, mean standard deviation of 1.89, and the effect size of 0.487.

For this purpose, 70 sound human molars that had been extracted within the past three months were disinfected using a 0.5% chloramine T solution at 4°C. Teeth were then kept in distilled water at 37°C for two weeks. The enamel in the occlusal surface of teeth was removed entirely with 180-grit sandpaper under water cooling with the circular polishing machine (Malekteb, Tehran, Iran) until the 7 mm diameter flat dentin was exposed. Molars were then embedded in brass molds of 2.5 × 3.5 cm filled with self-curing acrylic resin. 600-grit sandpaper was attached to the polishing machine used to standardize the smear layer under water lubrication. Specimens were randomly divided into 7 groups.  Table 1 shows an overview of the study groups, materials used, and application modes according to their instructions for use. The samples were stored in distilled water at 37°C until the experiments finished.

**Figure 1:**
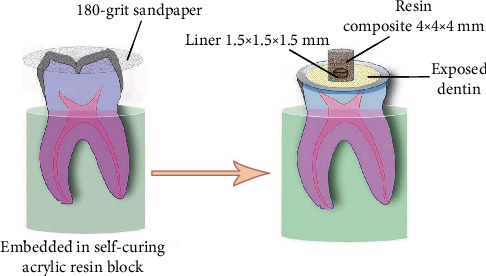
Representative scheme of sample preparation. (a) Removing the enamel of the occlusal surface with 180-grit sandpaper. (b) Applying liner and resin composite materials over the flat dentin area with the help of standardized polyethylene tubes.

The polyethylene tube was used to standardize the area and volume of liners (1.5 mm height and 1.5 mm internal diameter) and composite restorative materials (4 mm height and 4 mm internal diameter). The schematic picture of sample preparation is shown in Figure 1.

Caneppele et al. [7]. used bovine teeth for their study, and we could not use their dimensions. A pilot study was done with a different dimension of liners and composite; the results showed that this dimension was suitable for measuring shear bond strength, and the composite will be entirely on dentin.

After the application of respective lining materials, dentin surfaces and the liners in all study groups were then etched with 37% phosphoric acid gel for 15 seconds and rinsed. Two layers of total-etch Tetric N bond (Ivoclar/Vivadent, Schaan, Liechtenstein) were applied over the liner and surrounding dentin surface with a bristle brush. It was rubbed for 15 s followed by gentle air drying for approximately 5 s and cured for 10 s by a light-curing unit (Bluephase C8; Ivoclar Vivadent, USA) with a light intensity of 800 mW/cm^2^ at a standardized distance of 1 mm for 40 seconds. The power intensity was repeatedly measured using a radiometer (Bluephase Meter II, Ivoclar Vivadent, Schaan, Liechtenstein) and displayed adequate intensity levels (800 mW/cm^2^).

The polyethylene tube (4 mm height and 4 mm internal diameter) was placed over the lining material and filled with the resin composite (Gradia (GC, Japan)) using the incremental technique (two 2-mm increments), and each increment was cured with a curing unit for 40 seconds. The polyethylene tubes were removed with a sharp knife after the completion of the resin composite build-up.

The specimens then underwent thermocycling (Delta Tpo2, Nemo, Mashhad, Iran) for 5000 cycles between 5 and 55°C with a dwell and transfer time of 30 s. Shear bond strength testing was performed in a universal testing machine Zwick/Roell Z050 (Zwick/Roell, Ulm, Germany) with a 50 kg load cell at a crosshead speed of 1 mm/min until bond failure occurred.

The fractured test specimens were examined under a stereomicroscope (SZX 16; Olympus, Tokyo, Japan) at a magnification of ×25 to analyze the nature of the failure. The modes of failures were classified into three groups: cohesive (fracture entirely within the liner or resin composite), adhesive (fracture at the interface of the material and dentin), and mixed (a combination of cohesive and adhesive failure) [25].

The data of the SBS test were analyzed using one-way ANOVA, and pairwise comparisons of the groups were performed using the Tamhane post hoc test. All statistical analyses were accomplished with SPSS version 22 at a 0.05 level of significance.

## 3. Results

**Figure 2:**
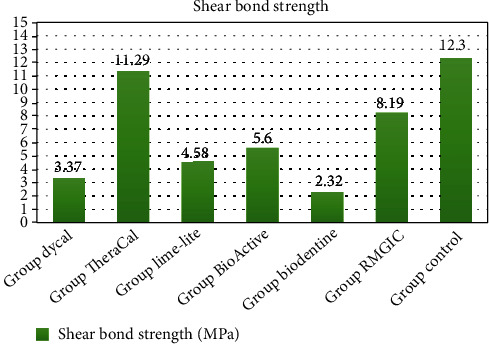
Comparative chart of shear bond strength results in different study groups.

According to the Kolmogorov-Smirnov test, all data presented a normal distribution (*P* > 0.05).  Table 2 shows the shear bond strength scores and standard deviations of the seven groups. Among the groups which received lining materials, TheraCal showed the highest shear bond strength (11.29 ± 1.89), followed by RMGIC (8.19 ± 4.71), and Biodentine showed the lowest shear bond strength (2.17 ± 1.29). The comparative chart of shear bond strength results of groups is shown in Figure 2.

According to pairwise comparisons of the groups (Table 2), the shear bond strength results among several groups are statistically significant (*P* < 0.05); among all 7 groups, only TheraCal did not significantly differ (*P* > 0.05) from the control (12.30 ± 1.54); other groups have a significant difference with the control group (*P* < 0.05). Also noteworthy is the fact that TheraCal had a significant difference with Dycal, Lime-Lite, Activa, and Biodentine. On the other hand, the difference in shear bond strength of Lime-Lite and Biodentine was only significant with TheraCal and control groups (*P* < 0.05). Another fact that needs to be highlighted is that the SBS of RMGIC was significantly higher than Biodentine and Dycal, groups that showed the lowest results of shear bond strength.

**Figure 3:**
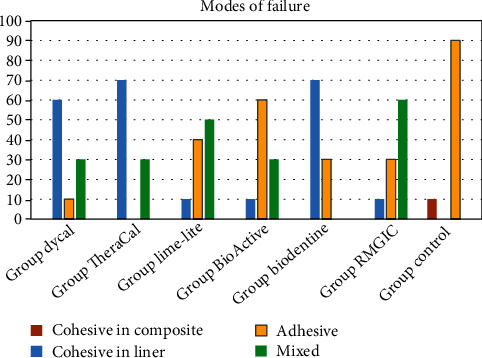
Frequency of different modes of failure in the groups.

The distribution of failure modes (Figure 3) of the specimens after the shear bond strength evaluation was characterized as adhesive, cohesive, or mixed. The observed modes of failure were predominantly cohesive in the respective pulp capping materials of Dycal, TheraCal, and Biodentine, while Activa showed more adhesive modes of failure. Mixed modes of failure were mostly shown in Lime-Lite and RMGIC specimens. No cohesive failure in the resin composite was observed.

## 4. Discussion

Since the pulp capping materials may affect the durability and condition of the tooth-restoration interface, the bond strength of these liners to dentin and restorative materials and their solubility during the etching process have impact on the success of restorations and also to maintain pulp vitality. This bond strength can be measured by various bond strength testing methods [2, 26]. Therefore, the present study was planned with the aim of evaluating and comparing the SBS of the liner and composite complex of six types of liners to the underlined tooth dentin structure.

Shear bond strength is the most commonly used technique to gauge bond strength and is used in approximately 30% of scientific papers worked on bond strength [22]. It is a simple, quick, and reliable method [23]. No further processing on the specimens is required after the bonding procedure [24]. In this study, the macro shear bond strength test was used since we wanted to evaluate a complex of liner and composite bond strength to dentin, which could not be evaluated by micro tests.

The results of SBS performed in the current study revealed that the highest bond strength was in the control (12.30 ± 1.54), followed by TheraCal (11.29 ± 1.89 MPa) and RMGIC (8.19 ± 4.71 MPa), which have significant differences with Biodentine (2.17 ± 1.29 MPa) and Dycal (3.37 ± 2.02 MPa) specimens showing the lowest shear bond strength. Lime-Lite (4.58 ± 2.21 MPa) and Activa (5.60 ± 1.93 MPa) also were significantly lower than theracal and control group. There was no significant difference between the Activa, Biodentine, Dycal, and Lime-Lite groups.

Biodentine exhibited significantly lower bond strength values as compared to other groups [27, 28]. In our study, the composite was placed over Biodentine after its initial setting time (12 min) as recommended by the manufacturer. According to previous studies [16, 29], it takes approximately 12 minutes for Biodentine to reach its initial setting time, while it needs as long as 14 days to get enough bulk strength to withstand the polymerization stresses. Consequently, the setting reaction of Biodentine may affect the bond strength between Biodentine and restorative materials. Furthermore, the exact mechanism of Biodentine's bonding to dentin is still not thoroughly clear. A combination of the chemical bond and a micromechanical anchorage provided by the infiltration of cement tags into the dentinal tubules are believed to be responsible for this bonding. The alkaline pH of Biodentine may cause the organic collagen component of the interfacial dentin to denature and become permeable, leading to intratubular tag formation combined with an interfacial mineral interaction layer called the mineral infiltration zone. Thus, in our study, placing the composite before the complete formation and maturation of the mechanical bond of Biodentine to dentin (after 12 minutes) could have resulted in lower SBS values [27, 28].

The other factor that can be attributed to the lower bond strength achived by Biodentine in our study may be due to the etching procedure over Biodentine. There are many studies that confirmed that etched Biodentine showed structural and chemical changes compared to nonetched Biodentine, which may affect the material's microhardness [11, 29–31]. However, all those studies mentioned that the etching process could negatively affect the shear bond strength of Biodentine to composite, while in this study, the SBS of the complex of liner-composite to dentin was measured. Thus, the solubility of water-based Biodentine under etching may contaminate the dentin surface bonding to the composite leading to the low SBS of the liner-composite complex to dentin. It seems that this process intensifies when the structure of Biodentine is not in its full maturation. This can also affirm the adhesive mode of failure found in Biodentine in the present study, because it can be due to the low bond of composite to the dentin surface surrounding the liner.

To overcome the limitation of poor bonding of calcium silicate materials in final restorations, TheraCal LC was introduced as a material for vital pulp therapy. In our study, TheraCal LC exhibited significantly higher bond strength values compared to other materials. This could be due to the fact that TheraCal is a resin-based light-curable liner with a high filler load (30%-50%) that can achieve sufficient early cohesive force upon photoactivation [25]; therefore, curing contraction of the overlying composite cannot cause stresses in the structure of TheraCal. The higher SBS of TheraCal may also be attributed to the hydrophilic resin-based methacrylate monomers of its structure that increase chemical adhesion to dentin and form a strong interface between TheraCal and the bonding surface [32, 33]. Furthermore, TheraCal has shown lower solubility than Biodentine and calcium hydroxide-based liners [3, 34, 35]; thus, it seems that the contamination of the dentin surface due to the etching process, which is supposed to have a role in the bonding of composite to dentin, is less likely to occur.

Theoretically, composites' bonding mechanism to calcium in liners is assumed to be comparable to the bonding mechanism that they have with the calcium of the tooth structure [12]. Compared to other pulp capping materials (except Biodentine), it has been proven that TheraCal LC releases a higher concentration of calcium ions, especially compared to Dycal and Lime-Lite [36]. Although the amount of calcium released by Lime-Lite, a light-cured resin-based calcium hydroxide liner, was less than the chemical-cured formula (Dycal) [37], the improved mechanical properties and presence of resin monomers in the structure of Lime-Lite led to higher SBS results than Dycal.

As mentioned, water resorption and solubility are two main physical properties of pulp capping material because degradation of the lining materials leads to the restoration's debonding and failure. In the study conducted by Gandolfi et al. [38], TheraCal showed lower solubility than Dycal, and Biodentine had the highest rates. It is perhaps because both Dycal and Biodentine are going through aging and etching stages; with both liners' high solubility properties in the water, lower SBS amounts were expected. Biodentine is prepared by mixing the mineral powder with water-based liquid, which is required to evaporate. Within the 12-minute setting time in our study and the 9 minutes in Gandolfi's, the water cannot be dried thoroughly, leading to a higher solubility rate. Moreover, they reported Lime-Lite as the less soluble material in water and acetic acid, among others. However, less solubility of light-cured calcium hydroxide liners in water due to the resin-based structure [3, 37] can justify better SBS results of Lime-Lite compared to Dycal; however, the difference was not significant.

As another reason, since Dycal lacks the resin content in its structure, unlike the TheraCal, its bond to resin composite is totally micromechanical, which means that penetration and interlocking of the adhesive systems into the surface irregularities play the main role in bonding [39].

In this present study, RMGIC (8.19 ± 4.71 MPa) got the second rank, among other materials, and did not significantly differ from TheraCal LC. Mehra et al. [14] conducted the study to measure the SBS of RMGIC with two different time intervals (immediately and 7 days) compared to TheraCal LC. They found that RMGIC showed higher SBS at a 7-day period (26.51 ± 1.05 MPa), even higher than TheraCal. This difference in results could be because, in the beginning, the RMGIC gets its high early strength by the polymerization of the methacrylate groups. After that, it is the acid-base reaction that helps the RMGIC to complete its setting and reach final strength [37]. Still, the fact that how long this acid-base reaction takes to complete is not precisely determined.

Activa is considered a dual-cured resin-modified glass ionomer (RMGI) and composed of bioactive glass as a filler and diurethane and methacrylate-based monomers with a modified polyacrylic acid and polybutadiene-modified diurethane dimethacrylate [40, 41]. Activa has desirable properties of glass ionomer cements plus resin-based materials simultaneously, such as water-friendly, releasing, and recharging fluoride and calcium phosphate ions, aesthetics, durability, and boosted physical properties [18].

It seems that Activa can release an equal amount of Ca and OH ions as TheraCal LC; therefore, it can be concluded that the ability of these two materials in stimulating the biomineralization of the tooth structure can be at the same level [42]; however, according to our results, SBS of Activa (5.60 ± 1.93 MPa) was significantly lower than TheraCal LC (11.29 ± 1.89 MPa) and also RMGIC (8.19 ± 4.71 MPa).

Although the structure of Activa BioActive products is similar to RMGICs, the laboratory and clinical findings report that the manufacturer's claim about the self-adhesive ability of this material has not been approved [43]. This assumption is in accordance with the study done by van Dijken et al. [44] in which a 1-year clinical evaluation of Activa BioActive posterior restorations indicated that when applied without using the adhesive as instructed by the manufacturer, Activa led to a nonacceptable very high failure rate after a year. It needs to be mentioned that, to the best of our knowledge, there is no data available about the solubility of Activa BioActive in water and acid; however, based on its composition, it can be inferred that Activa might have low solubility in water and acid; thus, acid etch might have a less destructive effect on its structure. Therefore, the lower values of Activa in the present study could be due to the absence of the dentin pretreatment procedure.

After the shear bond strength testing, failure modes were evaluated under a stereomicroscope and recorded as cohesive, adhesive, and mixed modes. Adhesive failure, which was predominantly found in Activa BioActive specimens, is an indicator of the lack of a strong bond at the lining material-composite/dentin interface due to not using an adhesive. In comparison, cohesive failures are more acceptable than adhesive failures because cohesive failure may be due to the material's low internal resistance or the bond strength being more higher than the material's internal resistance [45]. In our study, Dycal, TheraCal, and Biodentine showed more cohesive failure. Since Dycal and Biodentine are self-cured materials, this type of failure mode can be related to their low early bulk strength. TheraCal had more cohesive and a few mixed modes, which could also be due to its low bulk strength. On the other hand, as Mehra et al. [14] mentioned, this pattern might relate to the cohesive strength of material rather than the real weak bond strength in the interfaces.

The current study evaluated the SBS of the composite-liner complex to dentin in an in vitro situation; however, the oral cavity situation is different from the in vitro condition, leading to different material behaviors. Therefore, further in vivo investigations are required to obtain additional data. In order to evaluate the effect of the setting time on the adhesion of the Biodentine to the bonded surfaces or materials, future studies should consider measuring the mechanical properties by dedicating more time before the application of the final restorative material. As the type of the bonding material can have an influence on the SBS of composite to dentin, the behavior of these materials should be compared using other kinds of adhesives.

## 5. Conclusion

Within the limitation of this study, it can be concluded that

1. the bond strength of resin composite to dentin can be affected differently using various types of liners compared to not using them
2. TheraCal LC showed maximum shear bond strength when bonded with composite, and Biodentine showed the lowest shear bond strength values after its initial setting time (12 min)

## Figures and Tables

**Table 1:** Grouping of samples and materials used in the study.

Lining material (manufacturer) Batch number	Mode of application
Dycal (Dentsply Tulsa Dental, Johnson City, TN, USA) 170517	(1) Dispense equal volumes of base and catalyst pastes on the paper pad provided. (2) Stir immediately to mix thoroughly until a uniform color is achieved. (3) Complete mixing within 10 seconds. (4) Allow the liner to set (approximately 2-3 minutes) completely.
TheraCal LC (Pulpdent, Watertown, MA, USA) 1700002779	(1) Apply to the operatory area of the preparation. (2) Light cure for 20 s. (3) Apply the adhesive agent.
Lime-Lite (Pulpdent Corporation, Watertown, MA, USA) 160211	(1) Apply to the operatory area of the preparation. (2) Light cure for 20 s. (3) Apply the adhesive agent.
Activa BioActive BASE/LINER (Pulpdent, USA) 160211	(1) Remove cap so that base and catalyst are at the orifice of the syringe barrels. (2) Place a mixing tip on the automix syringe. (3) Dispense 1-2 mm onto a pad. (4) Dispense material directly onto the tooth surface and massage into the dentin for 20 seconds. (5) Light curing for 20 seconds.
Biodentine (Septodont, Saint Maur des Fosses, France) B19471	(1) Pour four drops from the liquid container into a capsule containing powder. (2) Close the capsule and mix for 30 s on a high-speed amalgamator. (3) Apply to operatory area. (4) Wait 12 min from the start of the mix to continue other steps.
RMGIC (Fuji II LC G.C., Europe N.V.) 1712071	(1) The standard powder to liquid ratio is 3.2 g/1.0 g (1 level scoop of powder to 2 drops of liquid). (2) Mixing (pull half of the powder onto liquid and mix with lapping strokes, pull in remaining powder, and mix thoroughly to a glossy consistency). (3) Light cure for 20 seconds.
Control (no liner)	Apply the resin composite after the application of adhesive on the prepared dentin surface.

**Table 2:** Mean shear bond strength scores (MPa) and standard deviations of the groups.

Group	Mean	Std. deviation	Minimum	Maximum
Dycal	3.3778^a^	2.02400	0.35	6.78
TheraCal	11.2938^bc^	1.89009	8.57	13.20
Lime-Lite	4.5888^ac^	2.21127	0.51	7.56
BioActive	5.6013^ac^	1.93269	2.47	8.34
Biodentine	2.3288^a^	1.29936	0.55	4.77
RMGIC	8.1963^c^	4.71168	1.06	16.64
Control	12.3050^bc^	1.54422	9.72	14.68

Different letters in the same column show statistically significant differences (*P* < 0.05).

## Data Availability

The data used to support the findings of this study are available from the corresponding author upon request.
